# Exposure to nicotine pouch marketing and nicotine pouch experimentation among U.S. adults who use commercial tobacco

**DOI:** 10.1016/j.pmedr.2024.102868

**Published:** 2024-08-23

**Authors:** Lilianna Phan, Kasra Zarei, Julia Chen-Sankey, Kiana Hacker, Aniruddh Ajith, Bambi Jewett, Kelvin Choi

**Affiliations:** a National Institute on Minority Health and Health Disparities Division of Intramural Research, Bethesda, MD, United States; b Department of Community Health and Prevention, Dornsife School of Public Health, Drexel University, Philadelphia, PA, United States; c Division of Graduate Nursing, College of Nursing and Health Professions, Drexel University, Philadelphia, PA, United States; d Institute for Nicotine & Tobacco Studies, Rutgers Biomedical and Health Sciences, New Brunswick, NJ, United States; e School of Public Health, Rutgers University, Piscataway, NJ, United States; f School of Medicine, University of Pittsburgh, Pittsburgh, PA, United States

**Keywords:** Nicotine, Nicotine pouch, Tobacco, Smokeless, Tobacco advertising, Tobacco marketing, Non-cigarette tobacco product

## Abstract

**Objective:**

U.S. nicotine pouch (NP) sales have grown since 2016. Among U.S. adults who currently and formerly used commercial tobacco, we examined demographic associations with learning about NPs through varying marketing channels, and their associations with NP experimentation overall and stratified by race/ethnicity.

**Methods:**

A nationally representative sample of U.S. adults who currently and formerly used commercial tobacco (n = 1,700) were surveyed online in January-February 2021. Participants reported their demographics and whether they learned about NPs through nine marketing channels. We used weighted multivariable logistic regressions to examine demographic associations of each channel, and each channel’s association with NP ever-use.

**Results:**

Overall, 45.2 % were aware of NPs, and 17.4 % ever used NPs. Highest reported NP awareness was through stores (16.6 %), internet/social media ads (9.2 %), friends/family’s social media (8.2 %), direct mail/email (7.6 %), and print media ads (6.9 %). Younger, female, Black and Hispanic (vs. White) adults had lower odds of NP awareness through various marketing channels than their counterparts. Black adults (vs. White) had higher odds of NP awareness through news stories on TV/radio/online. Awareness through each channel was associated with higher odds of ever-using NPs, especially brand sponsored events, brand website/social media accounts, and direct mail/email (p’s < 0.05). Results were generally consistent when stratified by race/ethnicity, but notable differences were also observed (race/ethnicity interaction p < 0.05).

**Conclusions:**

NP marketing may promote NP experimentation among U.S. adults who use commercial tobacco. Future research should examine whether exposure to NP marketing leads to poly-tobacco use, which could increase the detrimental health effects of tobacco use.

## Highlights

• Almost half of U.S. adults who use commercial tobacco were aware of nicotine pouches through various marketing channels.
• Awareness through each nicotine pouch marketing channel was associated with higher odds of nicotine pouch ever use.
• Nicotine pouch marketing may be equally reaching varying demographic segments of U.S. adults who use commercial tobacco.

## 1 Introduction

Nicotine pouches are a new class of smokeless tobacco products introduced to the market in 2016. Nicotine pouches are pre-filled, microfiber pouches that contain dissolving nicotine powder; they can be purchased in varying nicotine concentrations and flavors (Majmundar et al., 2022). Nicotine pouches differ from snus, which are cured and fermented tobacco processed into fine particles and packaged in ready-to-use pouches (Centers for Disease Control and Prevention, 2021). While health risks from varying tobacco products exist on a risk continuum (U.S. Food and Drug Administration, 2024), previous research has shown that nicotine pouches can have alarmingly high, and thus harmful, concentrations of nicotine and at times display misleading labeling about their nicotine content (Nadja et al., 2022). Nicotine may negatively affect the cardiovascular system, neurological development during adolescence and young adulthood, and fetal development during pregnancy (Mishra et al., 2015). Additionally, nicotine may drive the continuation and escalation of commercial tobacco use (Benowitz, 2009, Sung et al., 2018), the leading cause of preventable death worldwide (Samet, 2013). Those who use multiple tobacco products concurrently may also have an increased cumulative mortality risk (Choi et al., 2022).

Since 2016, nicotine pouch sales have rapidly increased more than 300-fold from 163,178 units ($709,635) to 45,965,455 units ($216,886,819) in 2020 (Marynak et al., 2021). The substantial increase in sales may be due largely in part to the industry’s marketing and advertising (Federal Trade Commission, 2023). A previous study found that approximately U.S. $18 million was spent on nicotine pouch marketing and advertising in the U.S. from January 2018 to April 2020 (Emery et al., 2022). Another previous study estimates that from January 2019 to September 2021, over U.S. $24 million was spent on total advertising expenditures for the nicotine pouch brands of Velo, Zyn, and On! alone (Duan et al., 2024). This previous study also identified that Velo, Zyn, and On! advertising expenditures prioritized online web-site display, print, radio, and television advertising channels (Duan et al., 2024). Using nicotine pouch expenditure data from 2016 to 2023, another previous study identified aired times for NP television and radio ads and found varying brands led nicotine pouch advertising expenditures for various marketing channels: Velo led the majority of television, radio, and mobile ads, Zyn led online display and video ads, and Black Buffalo led print ads (Ozga et al., 2024). While altogether these studies provide some insights on the industry’s advertising and marketing playbook for selling nicotine pouches, little is known about where and how individuals recall learning about nicotine pouches and how their exposure to various marketing channels for nicotine pouches may differ by demographic characteristics. This type of information is important for surveillance given the tobacco industry’s targeted marketing strategies that has shaped tobacco use disparities (Lee et al., 2015, U.S. National Cancer Institute, 2017, Moran et al., 2019). Furthermore, while previous research has found that exposure to tobacco product marketing is associated with its use (Choi et al., 2020, Hébert et al., 2023, Donaldson et al., 2022), little is known about how these varying marketing channels for learning about nicotine pouches may be related to use of nicotine pouches. Thus, we examined demographic associations with awareness of nicotine pouches by varying marketing channels among U.S. adults who currently and formerly used commercial tobacco products. We also examined associations of learning about nicotine pouches through each channel and its associations with ever use of nicotine pouches overall, and stratified by race and ethnicity, among this nationally representative sample of U.S. adults who currently and formerly used commercial tobacco products. Stratification by race and ethnicity is important given racial disparities in tobacco use (Lee et al., 2015, U.S. National Cancer Institute, 2017, Moran et al., 2019).

## 2 Materials and methods

### 2.1 Study design

Data were from a U.S. representative sample of adults (≥21 years) who currently and formerly used commercial tobacco (n = 1,700), with oversampling of self-identified Asian/Asian American and Black/African American adults from the YouGov online panel in January-February 2021. YouGov panel members are recruited from multiple sources, including standard advertising and partnerships with a wide range of websites. YouGov panel members who may be interested and meet eligibility criteria for this survey were invited by email to participate. The purpose of the overall study was to understand how COVID-19 and life experiences influence commercial tobacco use behaviors among U.S. adults who currently and formerly used commercial tobacco products. Commercial tobacco products include cigarettes, electronic cigarettes, cigars, little filtered cigars, cigarillos, filtered cigars, hookah tobacco, other combustible tobacco products (e.g., roll-your-own cigarettes, pipe, etc.), and smokeless tobacco. Current commercial tobacco use includes adults who use commercial tobacco every day or some days at the time of the survey. Former commercial tobacco use is captured as adults who have used commercial tobacco products in the past twelve months prior to the survey, but not at the time of the survey. YouGov used a sampling matching approach with weighting, derived from a number of sources including the U.S. Census, to achieve national representation of the target population similar to traditional random-digit dialing sampling (Rivers, 2009). Briefly, the matched sample was weighted to the sampling frame using propensity scores, based on key demographic variables. Post-stratification weighting was performed by deciles of propensity scores, and then trimmed and normalized to equal sample size. These post-stratification weights were applied in all analyses to achieve national representation of U.S. adults who currently and formerly used tobacco. Eligible individuals completed the online survey after providing informed consent and were compensated according to YouGov policy (cooperation rate = 88.3 % among those screened eligible). This study used deidentified data, which does not require review or approval from the Institutional Review Board per National Institutes of Health policy and 45 CFR 46.

### 2.2 Measures

#### 2.2.1 Demographics

Participants reported their age (collected and coded as a continuous variable), biological sex (coded as male or female), race and ethnicity (coded as Asian/Asian American, Black/African American, Latino/Hispanic [any race], White, and other race [including Middle Eastern or North African, Pacific Islander, Multiracial/Multiethnic, and “Other” race]), marital status (coded as has a partner [including living with someone in a marriage-like relationship, married] or no partner [including single never been married, divorced, separated, widowed, and other]), education level (coded as ≤high school or >high school), and annual household income level (coded as <$50,000 or ≥$50,000).

#### 2.2.2 Awareness (ever seen or heard) of nicotine pouches

For survey questions related to nicotine pouches, participants were first provided an image with four branded nicotine pouches commonly sold, including the packaging and nicotine pouches (i.e., Dryft, On, Velo, and Zyn) as examples of what nicotine pouches look like. Participants were asked to report whether they had seen or heard of nicotine pouches before this study. Participants reported yes, no, or not sure. Participants’ responses were recoded as yes and no/not sure.

#### 2.2.3 Marketing channels for awareness of nicotine pouches

Participants who reported ever seeing or hearing of nicotine pouches before this study were also asked how they learned about nicotine pouches. For each of the following marketing channels, participants were asked to respond yes or no: print media ads, internet/social media, direct mail/email, brand sponsored events, brand outdoor ads, brand website/social media account, friends and family’s social media, news stories on television/radio/online, or in stores. Participants who did not report ever seeing or hearing of nicotine pouches before this study were coded as not being aware of nicotine pouches through the individual marketing channels. We adapted this approach from previous tobacco-related studies (Stanton et al., 2022, Chen-Sankey et al., 2024) and included various NP marketing channels that have been previously identified in research (Duan et al., 2024, Hébert et al., 2023, Ling et al., 2023, Czaplicki et al., 2022) or as a longstanding tobacco industry marketing strategy (Gannon et al., 2023, O'Brien et al., 2020, Campaign for Tobacco-Free Kids, 2023). A composite count variable of the number of marketing channels through which a participant reported ever seeing or hearing of nicotine pouches was also created as previous research has shown each exposure to tobacco advertising is associated with increased odds of tobacco use (Hébert et al., 2023, Stanton et al., 2022).

#### 2.2.4 Ever use of nicotine pouches

In a single item, participants were asked to report their experience with nicotine pouches. Response options included “never used it before”, “used it before but not currently”, and “currently using it some days or every day.” Participants were not provided additional descriptions about these response options. Participant responses were recoded to characterize ever use (i.e., used it before but not currently, and currently using it some days or every day) and never use (i.e., never used it before).

### 2.3 Statistical analysis

Post-stratification weights were applied in all analyses to achieve national representation of U.S. adults who currently and formerly used tobacco. Weighted distributions of demographics, awareness through each marketing channel or any marketing channel, and ever use of nicotine pouches were estimated. The prevalence of awareness of nicotine pouches through each marketing channel by demographics was also estimated. Weighted multivariable logistic regression models were used to examine the associations between demographics (age, biological sex, race and ethnicity, marital status, education level, and income level) and awareness about nicotine pouches through each marketing channel. A zero-inflated Poisson regression model was used to examine the association between the same demographics and the count of marketing channels through which participants became aware of nicotine pouches. Weighted multivariable logistic regression models were used to examine the associations between: 1) awareness of nicotine pouches through each marketing channel and ever use of nicotine pouches, adjusting for age, biological sex, marital status, education level, income level, race/ethnicity, and with and without the interaction between each marketing channel and race/ethnicity; and 2) between the count of marketing channels and ever use of nicotine pouches, adjusting for age, biological sex, marital status, education level, and income level, and stratifying for race/ethnicity. We also added the interaction for race/ethnicity * marketing channel in the models and examined pairwise comparisons. Prevalence and model parameter estimates were calculated using the SAS SURVEYFREQ, SURVEYLOGISTIC, and GLIMMIX procedures incorporating weights, and parameter estimates were summarized implemented in SAS® software version 9.4 (SAS Institute: Cary, NC). Results with p < 0.05 were considered to be statistically significant.

## 3 Results

Weighted demographics and nicotine pouch awareness, marketing channels, and ever use are shown in Table 1. Over half of the U.S. adults who currently and previously used commercial tobacco were male (59.1 %; vs. female) with a sample mean age of 42.70 years (standard error = 0.57). The majority of them self-identified with White race (66.4 %), with smaller proportions of them self-identifying with Black/African American race (13.6 %), Latino/Hispanic ethnicity [any race] (13.1 %), Asian/Asian American race (4.5 %), and another race (i.e., Middle Eastern or North African, Pacific Islander, Multiracial/Multiethnic, and “Other” race]) (2.4 %), respectively. About half of these adults reported having a partner (53.6 %; vs. no partner). About equal proportions of them reported having ≤high school education (47.8 %) and >high school education (52.2 %), while 60.6 % reported an annual household income level of <$50,000 (vs. ≥$50,000).

**Table 1:** Demographic, nicotine pouch use, and nicotine pouch marketing channel distributions among U.S. adults who used commercial tobacco during the 12 months prior to the survey, 2021.

Variable	Unweighted *N*	Weighted %
**Age:** mean (SE) = 42.70 (0.57)	–	–

**Biological sex**		
Female	734	40.86
Male	966	59.14

**Race/ethnicity**		
Asian/Asian American	258	4.51
Black/African American	310	13.59
Latino/Hispanic	271	13.12
Other^1^	67	2.35
White	794	66.43

**Marital status**		
No partner	788	46.43
Has a partner	912	53.57

**Education level**		
≤High school	569	47.77
>High school	1131	52.23

**Income level**		
<$50,000	987	60.58
≥$50,000	711	39.42

**Awareness of nicotine pouches**		
Yes	752	45.23
No	948	54.77

**Ever use of nicotine pouches**		
Yes	297	17.44
No	1403	82.56

**Marketing channels of nicotine pouch awareness**		
In stores	240	16.60
Internet/social media ads	150	9.18
Friends and family’s social media	140	8.18
Direct mail/email	156	7.59
Print media ads	141	6.94
Brand outdoor ads	104	4.57
News stories on TV/radio/online	79	4.45
Brand website/social media account	78	3.17
Brand sponsored events	63	2.70
Any channel	588	35.89

Notes: Sample was weighted to the sampling frame using propensity scores, based on key demographic variables. Post-stratification weighting was performed by deciles of propensity scores, and then trimmed and normalized to equal sample size. These post-stratification weights were applied in all analyses to achieve national representation of U.S. adults who currently and formerly used tobacco.

1 “Other” race includes Middle Eastern or North African, Pacific Islander, Multiracial/Multiethnic, and “Other” race.

Overall, among U.S. adults who formerly and currently use commercial tobacco products, 45.2 % reported having ever seen or heard of nicotine pouches. About 35.9 % report learning about nicotine pouches through one or more of the marketing channels studied. Specifically, the largest proportions of them became aware of nicotine pouches through the following marketing channels: in stores (16.6 %), internet/social media ads (9.2 %), friends and family’s social media (8.2 %), direct mail/email (7.6 %), and print media ads (6.9 %). Proportions for other marketing channels are reported in Table 1.

Table 2, Table 3 report the weighted prevalence estimates of marketing channels for awareness of nicotine pouches by demographics and the demographic associations with each nicotine pouch marketing channel, respectively. Younger adults had lower odds of learning of nicotine pouches through internet/social media ads (adjusted odds ration [AOR] = 0.97, 95 % confidence interval [CI] = 0.94–0.99) and branded outdoor ads (AOR = 0.96; 95 % CI = 0.94–0.99) than those who are older adults. Female adults also had lower odds of learning of nicotine pouches in stores (13.4 % vs. 18.8 %; AOR = 0.65, 95 % CI = 0.42–0.98) compared to male adults. Black/African American (10.1 % vs. 20.4 %; AOR = 0.42, 95 % CI = 0.23–0.78) and Hispanic adults (8.5 % vs. 20.4 % AOR = 0.34, 95 % CI = 0.18–0.62) had lower odds of learning about nicotine pouches in stores than White adults, whereas Black/African American adults (9.4 % vs. 3.8 % AOR = 2.68, 95 % CI = 1.09–6.63) had higher odds of learning about nicotine pouches in news stories in television, radio, or online than White adults. Additionally, younger adults (AOR = 0.98, 95 % CI = 0.97–0.99) had lower odds of learning about nicotine pouches through any marketing channel than their counterparts. Furthermore, there were comparable associations between demographics and the count of marketing channels through which participants became aware of nicotine pouches with younger (adjusted relative risk [ARR] = 0.99, 95 % CI = 0.98–0.99) and female (ARR=0.83, 95 % CI = 0.71–0.98) adults having a lower risk of learning about nicotine pouches through the marketing channels surveyed than those who are older, male, and White adults respectively (Supplemental Table 1).

**Table 2:** Weighted prevalence of nicotine pouch marketing channels for nicotine pouch awareness by demographics among U.S. adults who used commercial tobacco during the 12 months prior to the survey, 2021.

Variables	Marketing Channels for Awareness of Nicotine Pouches (weighted prevalence %):									Any Channel
	In stores	Internet/social media ads	Friends and family’s social media	Direct mail/email	Print media ads	Brand outdoor ads	News stories on TV/radio/online	Brand website/social media account	Brand sponsored events	
**Biological sex**										
Female	13.4	8.3	9.2	6.6	6.8	3.2	4.3	3.5	1.8	33.1
Male	18.8	9.8	7.5	8.3	7.0	5.5	4.6	3.0	3.3	37.8

**Race/Ethnicity**										
Asian/Asian American	4.9	7.2	12.0	5.1	10.9	6.2	3.2	3.8	6.7	33.5
Black/African American	10.1	8.9	7.6	4.8	6.6	2.3	9.4	5.0	4.0	30.6
Latino/Hispanic	8.5	11.8	6.7	7.5	9.5	7.0	2.9	2.7	3.8	32.9
Other^1^	13.7	10.6	6.9	10.1	4.2	1.8	4.1	2.1	6.4	34.3
White	20.4	8.8	8.4	8.3	6.3	4.5	3.8	2.9	1.8	37.8

**Marital Status**										
No Partner	17.1	7.7	8.3	7.0	8.6	3.9	4.5	3.5	2.2	34.2
Has a Partner	16.1	10.5	8.0	8.1	5.5	5.2	4.4	2.9	3.2	37.4

**Education Level**										
≤High school	15.6	10.0	9.1	7.2	6.7	3.9	4.4	2.9	3.0	38.5
>High school	17.5	8.4	7.4	7.9	7.2	5.2	4.5	3.4	2.4	33.5

**Income Level**										
<$50,000	17.1	8.1	7.8	7.5	7.4	4.9	4.5	3.3	3.2	35.7
≥$50,000	15.9	10.8	8.7	7.8	6.3	4.1	4.4	3.1	2.0	36.4

1 “Other” race includes Middle Eastern or North African, Pacific Islander, Multiracial/Multiethnic, and “Other” race.

**Table 3:** Demographic associations with marketing channels for nicotine pouches awareness among U.S. adults who used commercial tobacco during the 12 months prior to the survey, 2021.

Variables	Marketing Channels for Awareness of Nicotine Pouches (AOR [95 % CI]):								
	In stores	Internet/social media ads	Friends and family’s social media	Direct mail/email	Print media ads	Brand outdoor ads	News stories on TV/radio/online	Brand website/social media account	Brand sponsored events
**Age**	0.99 (0.98–1.01)	0.97 (0.94–0.99)	0.98 (0.97–1.00)	0.99 (0.98–1.01)	0.99 (0.98–1.01)	0.96 (0.94–0.99)	1.00 (0.98–1.02)	0.98 (0.96–1.00)	0.97 (0.93–1.00)

**Biological sex**									
Female	0.65 (0.42–0.98)	0.85 (0.50–1.43)	1.33 (0.76–2.34)	0.76 (0.45–1.31)	0.99 (0.60–1.63)	0.54 (0.28–1.03)	0.89 (0.45–1.79)	1.18 (0.58–2.39)	0.48 (0.20–1.16)
Male	REF	**–**	**–**	**–**	–	–	–	–	–

**Race/Ethnicity**									
Asian/Asian American	0.18 (0.08–0.39)	0.65 (0.32–1.30)	1.39 (0.57–3.37)	0.55 (0.28–1.09)	1.66 (0.63–4.38)	1.17 (0.54–2.54)	0.81 (0.31–2.09)	1.12 (0.48–2.60)	3.75 (0.95–14.79)
Black/African American	0.42 (0.23–0.78)	1.08 (0.51–2.30)	0.85 (0.40–1.79)	0.58 (0.26–1.31)	0.95 (0.46–1.96)	0.53 (0.21–1.30)	2.68 (1.09–6.63)	1.74 (0.61–4.96)	2.35 (0.86–6.43)
Latino/Hispanic	0.34 (0.18–0.62)	1.18 (0.59–2.37)	0.68 (0.27–1.67)	0.87 (0.44–1.70)	1.42 (0.66–3.05)	1.42 (0.67–3.03)	0.76 (0.32–1.81)	0.82 (0.36–1.87)	1.91 (0.67–5.42)
Other^1^	0.63 (0.16–2.46)	1.06 (0.26–4.34)	0.71 (0.12–4.05)	1.25 (0.24–6.61)	0.66 (0.15–3.04)	0.37 (0.10–1.36)	1.09 (0.22–5.33)	0.71 (0.20–2.50)	3.40 (0.80–14.36)
White	REF	–	–	–	–	–	–	–	–

**Marital Status**									
No Partner	1.14 (0.74–1.74)	0.87 (0.51–1.49)	1.13 (0.65–1.96)	0.87 (0.51–1.49)	1.56 (0.89–2.74)	0.62 (0.31–1.23)	0.95 (0.44–2.07)	1.17 (0.58–2.36)	0.48 (0.20–1.17)
Has a Partner	REF	–	–	–	–	–	–	–	–

**Education Level**									
≤High school	0.87 (0.55–1.38)	1.29 (0.72–2.32)	1.41 (0.77–2.61)	0.91 (0.52–1.59)	0.89 (0.52–1.54)	0.65 (0.33–1.28)	0.94 (0.46–1.92)	0.82 (0.36–1.85)	1.00 (0.46–2.14)
>High school	REF	–	–	–	–	–	–	–	–

**Income Level**									
<$50,000	1.14 (0.71–1.84)	0.68 (0.36–1.26)	0.77 (0.40–1.51)	1.05 (0.61–1.80)	1.10 (0.61–2.01)	1.57 (0.79–3.12)	0.95 (0.46–1.96)	0.98 (0.40–2.42)	1.89 (0.91–3.92)
≥$50,000	REF	–	–	–	–	–	–	–	–

Notes: Outcome for each exposure was modeled separately. Adjusted for biological sex, age, marital status, education level, and income level.

1 “Other” race includes Middle Eastern or North African, Pacific Islander, Multiracial/Multiethnic, and “Other” race.

Table 4 shows associations between each marketing channel for NP awareness and ever use of nicotine pouches stratified by race/ethnicity. Overall, learning about nicotine pouches through each of the marketing channels was associated with ever use of nicotine pouches (AOR range = 2.51–18.01) compared to not learning about nicotine pouches through the marketing channel. Results were relatively consistent across all racial and ethnic categories included in this study; for instance, learning about nicotine pouches through direct/email (AOR range = 4.09–123.74), brand outdoor ads (AOR range = 3.71–16.17), and in stores (AOR = 2.43–7.56) were associated with a greater likelihood of ever using nicotine pouches (vs. not learning about nicotine pouches through the marketing channel). We also regressed ever using nicotine pouches on the interaction of individual marketing channels with race and ethnicity, finding significant interactions with race/ethnicity and internet/social media ads for Asian/Asian American (Asian * internet social interaction test p = 0.005) and Black/African American adults (Black/African American * internet social interaction test p = 0.003) relative to White adults, and direct mail/email with significant interaction for Asian/Asian American adults (Asian * direct mail/email interaction test p < 0.0001) relative to White adults.

**Table 4:** Associations of marketing channels for awareness of nicotine pouches with ever use of nicotine pouches, stratified by race/ethnicity, among U.S. adults who used commercial tobacco during the 12 months prior to the survey, 2021.

Nicotine pouch marketing channels	Race/ethnicity (AOR [95 % CI])				
	Overall	Asian/Asian American	Black/African American	Latino/Hispanic	White
In stores	2.98 (1.75–5.05)	6.50 (1.52–27.84)	7.56 (1.73–32.94)	6.96 (2.15–22.61)	2.43 (1.30–4.53)
Internet/social media ads	2.51 (1.29–4.90)	16.87 (5.04–56.42)^1^	12.86 (3.44–47.99)^1^	3.44 (1.06–11.16)	1.54 (0.61–3.90)
Friends and family’s social media	5.13 (2.88–9.15)	1.36 (0.28–6.69)	10.42 (2.91–37.33)	1.77 (0.42–7.55)	6.90 (3.27–14.55)
Direct mail/email	6.12 (3.02–12.41)	123.74 (30.51–501.86)^2^	12.11 (3.42–42.80)	12.28 (4.14–36.42)	4.09 (1.57–10.64)
Print media ads	2.52 (1.35–4.68)	3.27 (0.84–12.73)	11.81 (2.91–47.93)	2.93 (0.96–8.97)	1.60 (0.70–3.66)
Brand outdoor ads	5.16 (2.57–10.33)	11.74 (3.34–41.25)	16.17 (2.31–113.34)	8.25 (2.63–25.86)	3.71 (1.37–10.08)
News stories on TV/radio/online	4.71 (2.23–9.92)	16.98 (3.47–83.04)	2.47 (0.33–18.43)	4.22 (1.21–14.76)	4.43 (1.63–12.10)
Brand website/social media account	17.81 (7.47–42.48)	69.95 (11.65–419.93)	40.85 (7.18–232.43)	7.54 (0.95–60.13)	14.97 (4.49–49.90)
Brand sponsored events	18.01 (7.14–45.43)	–	–	18.23 (4.34–76.58)	8.16 (2.42–27.47)
Count of marketing channels	2.20 (1.79–2.71)	4.63 (2.25–9.53)	3.68 (2.25–6.03)	2.58 (1.91–3.48)	1.88 (1.43–2.47)

Notes: Outcome for each exposure was modeled separately. Adjusted for biological sex, age, race/ethnicity (for the “overall” model), marital status, education level, and income level. Statistical interactions between race/ethnicity and each marketing channel were tested in the overall model to discern significant differences by race/ethnicity.

1 Indicates significantly different from White adults at p < 0.01.

2 Indicates significantly different from White adults at p < 0.0001.

## 4 Discussion

This study provides insights on *how and where* U.S. adults who currently and formerly use commercial tobacco products are learning about nicotine pouches, a newer type of tobacco product in the market. This type of surveillance research is important to understand the reach of nicotine pouch marketing given the dynamic commercial tobacco landscape and overall health toll associated with tobacco use (U.S. Department of Health and Human Services, 2014) and its disparities (Lee et al., 2015, U.S. National Cancer Institute, 2017, Moran et al., 2019). Therefore, this study examined the demographic correlates of nicotine pouch marketing channels and each marketing channel’s association with nicotine pouch experimentation (i.e., ever use), stratified by race and ethnicity. Overall, a large proportion of U.S. adults who currently and formerly used commercial tobacco reported learning about nicotine pouches in stores. Other predominant nicotine pouch marketing channels included through advertising on social media, friends and family’s social media accounts, and nicotine pouch marketing through direct mail and email marketing channels. These findings are consistent with previous studies’ that found large proportions of their samples learning and/or purchasing nicotine pouches in convenience stores (Marynak et al., 2021), tobacco retailers and gas stations, in addition to through their friends, family, and co-workers (Tosakoon et al., 2023).

As found in this present study, previous research also noted that their U.S. young adults learned about nicotine pouches through direct mail and email marketing (Tosakoon et al., 2023). Monitoring of direct mail/email marketing is particularly important given that this marketing channel has been used by the tobacco industry as a way to circumvent previous marketing restrictions for other tobacco products (Silver et al., 2022, Henriksen, 2012). Direct mail/email tobacco marketing is also strongly associated with tobacco use (Choi and Forster, 2014). Taken altogether, these nicotine pouch marketing channels align with the longstanding practices of the tobacco industry in rolling out their tobacco products in the marketplace (U.S. Institute of Medicine Committee on Preventing Nicotine Addiction in Children and Youths, 1994). U.S. adults becoming aware of nicotine pouches through friends and family’s social media may also suggest potential trickle-down effects of direct marketing, which are designed to help shape attitudinal and risk perceptions about a tobacco product and normalize its use (U.S. Institute of Medicine Committee on Preventing Nicotine Addiction in Children and Youths, 1994).

While there were some varying prevalence estimates for learning about nicotine pouches through the nine marketing channels included in this study by demographic characteristics, there were few demographic correlates identified with each marketing channel (as shown in Table 2). These findings suggest that nicotine pouch marketing is equally reaching each demographic segment of the adult population who use commercial tobacco. Future research should continue to monitor nicotine pouch marketing exposure by subpopulations and potential demographic associations with these marketing channels. Marketing practices may evolve as the tobacco industry continues to position nicotine pouches in the market. Future research is needed to also better understand *what* information about nicotine pouches people are hearing, seeing, and sharing, and how repeated exposure to these messages may influence behaviors. These areas of research will also be important among U.S. adults who have not used commercial tobacco products.

We found consistently that exposure to nicotine pouch marketing through the studied marketing channels was associated with nicotine pouch experimentation among U.S. adults who currently and formerly use commercial tobacco products. These findings may imply the influence of tobacco industry marketing on use behaviors, as previously shown with marketing for cigarettes, e-cigarettes, and cigars and their use (Campaign for Tobacco-Free Kids, 2023, U.S. Department of Health and Human Services, 2014, Tosakoon et al., 2023, Silver et al., 2022, Henriksen, 2012). Additionally, given our findings on associations of marketing channels and ever-using nicotine pouches when stratified by race and ethnicity, some of these strategies (e.g., Internet/social media ads, direct mail/email marketing) may be particularly influential among racial/ethnic minority populations (e.g., Asian/Asian American and Black/African American adults), though Asian/Asian American and Black/African American adults who currently and formerly used commercial tobacco were not disproportionately exposed to these nicotine pouch marketing channels. Notably, tobacco marketing in these channels is the least regulated as compared to other channels (e.g., brand outdoor ads, print media ads) (Henriksen, 2012). Consequently, our findings on associations of marketing channels and ever-using nicotine pouches when stratified by race and ethnicity may suggest that if current regulation on tobacco marketing is simply extended to NP marketing, Asian/Asian American and Black/African American adults may be particularly vulnerable to the least regulated marketing channels (i.e., direct mail/email and internet/social media ads) (Moran et al., 2019). Future research is needed to better understand why U.S. adults who currently and formerly use commercial tobacco products may be using nicotine pouches, and how marketing strategies influence co-use of tobacco products or replacement. Future research should also consider examining the context with how exposure to a particular nicotine pouch marketing channel may lead to experimentation (e.g., brand sponsored events to nicotine pouch use). These areas of future research will inform public health efforts aiming to prevent and reduce the risks of poly-tobacco use.

There are limitations to this study. Findings from this nationally representative sample of U.S. adults who currently and formerly used commercial tobacco products may not generalize to younger populations in the U.S. and those who have not used commercial tobacco. While there is a potential that participants may report one marketing channel exposure as multiple marketing channel exposures, this limitation is unlikely given the distinctions between the varying marketing channels included in this study (e.g., direct email/mail, print media ads). Additionally, some results include wider confidence intervals due to smaller sample sizes (e.g., for those with awareness of nicotine pouches through each marketing channel, race/ethnicity stratification) although associations were generally consistent across each marketing channel and when stratified by race/ethnicity. We were also unable to examine nicotine pouch marketing channels by participants’ geographical characteristics (e.g., metropolitan vs. rural residency), which may influence their exposure to nicotine pouch marketing channels. Future research is needed to continue to monitor these associations and to examine how populations not included in this study are learning about nicotine pouches and nicotine pouch perceptions and behaviors. For example, future research should examine potential demographic correlates among specific priority populations including U.S. young adults ≤21 years old, those who do not use commercial tobacco, and sexual and gender minoritized populations that we were not able to examine in this study. Additionally, this research focuses on various NP marketing channels that could fall under regulatory authority for surveillance and monitoring. Future research should consider additional channels for how individuals learn about nicotine pouches or newer tobacco products. Finally, because this is a cross-sectional study, the directionality of the associations between NP marketing channel exposure and NP use needs to be confirmed by future longitudinal studies.

### 4.1 Conclusions

While we found some variations between groups when examining demographic associations with NP marketing channels examined in this study, findings from this nationally representative study suggest that nicotine pouch marketing channels may be reaching each segment of the population studied equally overall. Findings also suggest that awareness of NP through marketing channels may promote nicotine pouch experimentation among U.S. adults who use commercial tobacco products, which were generally consistent by race and ethnicity. Future research is needed to continue to monitor nicotine pouch marketing channels and to understand how nicotine pouch marketing may influence nicotine pouch use behaviors.

## Funding

This research was supported by the National Institute on Minority Health and Health Disparities Division of (NIMHD DIR), 10.13039/100000002National Institutes of Health (NIH) Intramural Research Program. Lilianna Phan was supported by NIMHD DIR, the Pathway to Independence Award in Tobacco Regulatory Research from National Cancer Institute (NCI)/FDA (R00CA272919), NCI Health Disparities Research Loan Repayment Program (L60CA294397), and the NIH FIRST Program (U54CA267735), with funding support from the Office of Director (OD), NIH. Julia Chen-Sankey was supported by the Pathway to Independence Award in Tobacco Regulatory Science from NCI/FDA (R00CA242589), Penn/Rutgers TCORS (U54CA229973), and Rutgers Cancer Institute of New Jersey Cancer Center Support Grant (P30CA072720). NIMHD DIR and NIH/FDA had no role in the study design, collection, analysis or interpretation of the data, writing the manuscript, or the decision to submit the paper for publication. The content was not reviewed by the FDA but underwent the standard manuscript clearance process for scientific papers published from the NIH Intramural Research Program. The opinions and comments expressed in this paper belong to the authors and do not necessarily reflect those of the U.S. Government, Department of Health and Human Services, National Institutes of Health, and National Institute on Minority Health and Health Disparities.

## CRediT authorship contribution statement

**Lilianna Phan:** Writing – original draft, Validation, Conceptualization. **Kasra Zarei:** Writing – review & editing, Visualization, Formal analysis, Conceptualization. **Julia Chen-Sankey:** Writing – review & editing, Visualization, Conceptualization. **Kiana Hacker:** Writing – review & editing, Visualization, Conceptualization. **Aniruddh Ajith:** Writing – review & editing, Visualization, Conceptualization. **Bambi Jewett:** Writing – review & editing, Visualization, Conceptualization. **Kelvin Choi:** Writing – review & editing, Visualization, Supervision, Funding acquisition, Conceptualization.

## Declaration of competing interest

The authors declare that they have no known competing financial interests or personal relationships that could have appeared to influence the work reported in this paper.

## Data Availability

Data will be made available on request.
