# Supplementary material for: Exposure to nicotine pouch marketing and nicotine pouch experimentation among U.S. adults who use commercial tobacco
Source: Prev Med Rep. 2024 Aug 23;46:102868. doi: 10.1016/j.pmedr.2024.102868 (PMC11399598; doi:10.1016/j.pmedr.2024.102868)
Supplement: Supplementary Data 1 [file mmc1.docx]

**Supplemental Table 1.** Zero-inflated Poisson regression of demographics with marketing channel counts for nicotine pouch awareness among U.S. adults who used commercial tobacco during 12 months prior to the survey, 2021.

| **Variables** | **Relative Risk (ARR [95% CI]):** |
| --- | --- |
| **Age** | 0.987 (0.98-0.99) |
| **Biological sex** |  |
| Female | 0.83 (0.71-0.98) |
| Male | REF |
| **Race/Ethnicity** |  |
| Asian/Asian American | 0.97 (0.72-1.29) |
| Black/African American | 0.84 (0.67-1.06) |
| Latino/Hispanic | 0.94 (0.75-1.18) |
| Other^1^ | 0.89 (0.56-1.42) |
| White | REF |
| **Marital Status** |  |
| No Partner | 0.93 (0.79-1.09) |
| Has a Partner | REF |
| **Education Level** |  |
| ≤ High school | 0.92 (0.77-1.09) |
| > High school | REF |
| **Income Level** |  |
| < $50,000 | 1.11 (0.93-1.32) |
| ≥ $50,000 | REF |

^1^“Other” race includes Middle Eastern or North African, Pacific Islander, Multiracial/Multiethnic, and “Other” race.

Notes: Adjusted for age, sex, race/ethnicity, marital status, education level, and income level.
